# Dual Biologic Therapy in Patients with Rheumatoid Arthritis and Psoriatic Arthritis

**DOI:** 10.5041/RMMJ.10494

**Published:** 2023-04-30

**Authors:** Victoria Furer, Ori Elkayam

**Affiliations:** 1 Department of Rheumatology, Tel Aviv Sourasky Medical Center, Tel Aviv, Israel; 2 Sackler Faculty of Medicine, Tel Aviv University, Tel Aviv, Israel

**Keywords:** Biological drugs, dual biological therapy, rheumatoid arthritis, psoriatic arthritis, safety

## Abstract

Treatment with biological agents has become standard of care in treatment of immune-mediated diseases (IMD), including rheumatoid arthritis and psoriatic arthritis. Yet, a significant proportion of patients experience loss of response to biologics, need treatment escalation, or develop side effects. During the past decade, new biologic agents with different targeted molecular pathways have been approved for treatment of IMD, introducing the possibility of concomitant dual biologic therapy. The role of dual biologic therapy targeting different inflammatory pathways has become an area of great interest in the field of IMD, addressing the unmet clinical need of patients with refractory diseases and treatment of comorbidities, such as osteoporosis, asthma, atopic dermatitis, and urticaria. Despite the increasing use of biologics as a dual therapy across different indications, there is a paucity of data concerning the safety of the simultaneous use of more than one biological agents. The purpose of this review is to summarize the current literature on the use of dual biologics in patients with rheumatoid arthritis and psoriatic arthritis, addressing the potential adverse effects associated with combination therapy, and highlighting future directions in the use of this novel therapeutic modality.

## INTRODUCTION

The advent of biologic therapies has revolutionized the treatment approach and outcomes in the field of chronic immune-mediated diseases (IMD), leading to relief of symptoms and delay of disease progression. While most patients with IMD achieve adequate disease control with biologic monotherapy or through combination with oral immunosuppressants, certain patients fail to respond, representing a therapeutic challenge. The biologic treatment armamentarium for rheumatoid arthritis (RA) and psoriatic arthritis (PsA) includes anti-cytokine biologics such as tumor necrosis factor inhibitor (TNFi), interleukin (IL)-1 inhibitor (IL-1i), IL-6 inhibitor (IL-6i), IL-17 inhibitor (IL-17i), IL-12/23 inhibitor (IL-12/23i), and IL-23 inhibitor (IL-23i); T-cell co-stimulation inhibitor (CTLA4-Ig) and B-cell depleting agents are also used. In clinical practice, biologic treatment is used sequentially and switched in cases of therapeutic resistance or intolerance.^1^,^2^ Despite a wide range of biologics with different mechanisms of action, less than half of RA patients achieve sustained remission, and up to 15% are refractory to treatment.^3^ Similarly, a substantial proportion of PsA patients fail to reach remission and thus face living with residual disease.^4^ Therefore, refining treatment strategies is required to optimize the care and reduce the burden of disease in resistant cases. Concomitant neutralization of multiple inflammatory pathways might offer a promising treatment approach in this setting. The rationale for the clinical use of dual biologic therapy is based on animal models that showed additive synergistic effect of dual therapy with IL-1 receptor antagonist and PEGylated soluble TNF receptor type I in rats with adjuvant-induced and collagen-induced arthritis.^5^,^6^ In both studies, dual biologic therapy achieved a higher efficacy than either agent alone in reducing the severity of arthritis and joint destruction. In the last two decades, the role of dual biologic therapy targeting different inflammatory pathways has become an area of great interest in the field of resistant IMD. Several randomized controlled clinical trials (RCTs) investigated the efficacy and safety of dual biologic treatment in RA with conflicting results,^7^–^12^ raising an important safety alert. In PsA, limited information concerning efficacy and safety of dual biologic treatment approach is available based on case reports or retrospective case series.^13^,^14^

The second clinical setting of dual biologic therapy use in patients with IMD is related to the concurrent treatment of comorbidities, such as advanced osteoporosis, severe bronchial asthma, atopic dermatitis, and urticaria. Osteoporosis is the most prevalent comorbidity reported in up to 25% of RA patients^15^ and in some patients with PsA.^16^ Dual biologic therapy based on a combination of biologic disease-modifying antirheumatic drugs (bDMARD) and denosumab (an anti-RANK-ligand monoclonal antibody used as a second-line treatment for osteoporosis) was reported in a number of retrospective^17^ and observational studies^18^,^19^ with favorable safety profile. Concerning the use of dual biologic therapy for other comorbidities, only limited information is available in the form of case reports.

The goal of this review is to summarize the current literature on the efficacy and safety of dual biologics in RA and PsA, addressing the potential adverse effects associated with combination therapy, and highlighting the future directions for use of dual biologics in patients with IMD.

## RHEUMATOID ARTHRITIS

### Dual Biologic Therapy Indicated for Refractory Disease

Following the success of preclinical studies of dual biologic therapy in animal models of arthritis,^5^,^6^ Genovese et al. conducted a pioneer trial in RA to evaluate the synergistic effects of combination therapy with the selective anti-TNFi biologic etanercept and the IL-1i anakinra.^7^ This randomized controlled trial (RCT) included biologic-naïve patients with active RA (*n*=244) despite methotrexate (MTX) therapy randomized to etanercept only (25 mg twice weekly), full-dosage etanercept (25 mg twice weekly) plus anakinra (100 mg/day), or half-dosage etanercept (25 mg once weekly) plus anakinra (100 mg/ day) treatment for 6 months. In contrast to the study hypothesis, the combination therapy with etanercept plus anakinra provided no treatment benefit over etanercept alone, regardless of the regimen, but was associated with an increased safety risk. The incidence of serious infections, injection-site reactions, and neutropenia was increased with combination therapy. Notably, there was a dose-dependent increase in the rate of serious infections with 0%, 3.7%, and 7.4% in patients treated with etanercept alone, half-dose etanercept with anakinra, and full-dose etanercept with anakinra, respectively. Weinblatt et al. evaluated the safety of intravenous abatacept (10 mg/kg) added to a background of non-biologic and biologic agents (TNFi and IL-1i) in patients with RA with a one-year follow up (ASSURE trial).^8^ Among the study cohort, a total of 103 patients received dual biologic therapy. Serious adverse events (SAEs) occurred more frequently in this subgroup (22.3%) than in other subgroups (11.7%–12.5%). Serious infections were observed in 5.8% of patients on dual biologic therapy compared to 2.6% of those on abatacept alone. Consistently with the previous study results, the post-hoc analysis failed to show any clinical benefit of dual biologic therapy. In 2007, Weinblatt et al. further investigated the efficacy and safety of addition of intravenous abatacept (2 mg/kg) to etanercept (50 mg/wk) in patients with active RA (*n*=121) during a 1-year RCT.^9^ This trial failed to demonstrate clinical benefit of the dual biologic therapy evaluated at 6 and 12 months of treatment, with an exception of a significantly higher response rate (the American College of Rheumatology [ACR] score ACR70) in the dual biologic versus single biologic group at 6 months. Yet, the interpretation of the efficacy results in this trial is limited in view of the use of a suboptimal dose of abatacept and early discontinuation of enrollment due to the shortage of etanercept supply. In terms of safety, the overall frequencies of adverse events were comparable between groups at 6 months, whereas, at 1 year, patients on dual biologic therapy had higher frequencies of adverse events and related study discontinuation, SAEs (16.5% versus 2.8%), and serious infections (3.5% versus 0%) compared to the placebo and etanercept groups. It is important to note that in these studies some patients continued background MTX, other non-biologic DMARD therapy, and/or concomitant corticosteroids, while being treated with dual biologic therapy, which may increase the risk of adverse events. Based on the RCTs results,^7^–^9^ the official guidelines from the ACR on the treatment of RA advised against the dual biologic therapy in view of the adverse benefit–risk ratio.^20^

Despite the initially discouraging results, evolving evidence supported some clinical benefit and adequate safety profile of a dual biologic regimen based on rituximab (RTX).^10^–^12^ Blank et al. were the first to report a retrospective analysis of particularly resistant patients with long-standing RA treated with a combination of RTX and TNFi (etanercept) *(n*=6) compared with RTX treatment (*n*=12).^10^ In this small study, dual biologic therapy was both safe and effective in patients with severe RA. Furthermore, according to a case report, two patients with refractory RA were treated with a dual RTX and etanercept therapy which led to disease remission and was well tolerated.^21^ Greenwald et al. conducted a small RCT (*n*=51) to assess the safety of RTX in combination with TNFi (etanercept or adalimumab) and MTX in patients with active RA (TAME study).^11^ In this study, patients received one course of RTX (1 g). The incidence of serious infections through week 24 was low and did not significantly differ between the study groups. The safety profile of RTX combined with TNFi was consistent with the RTX safety profile in combination with MTX previously reported in RCTs using the approved dose of RTX (2 g).^22^,^23^ Although this study was not powered to test for efficacy, there was no clear evidence of efficacy advantage in patients receiving RTX in combination with TNFi and MTX. In view of the limited study size and follow-up and the use of a relatively low RTX dose, no firm conclusions concerning treatment efficacy could be drawn. Rigby et al. consistently reported a similar safety profile of dual biologic therapy including low-dose RTX (1 g) with either TNFi or abatacept in an open-label trial (SUNDIAL II study), including 176 patients with longstanding resistant RA.^12^ The SAE rate was similar over 48 weeks (22.4 events/100 patient-years, 95% CI 15.9–31.5). Efficacy responses improved numerically at week 48 compared with week 24, yet the interpretation of the efficacy outcomes should be limited in the absence of a placebo-controlled arm and other design-related limitations.

Most recently, a novel therapeutic approach to RA treatment utilizing the dual biologic therapy of TNFi with IL-17i was investigated in two clinical trials.^24^,^25^ The use of IL-17i in the treatment of RA was based on the significant increases in circulating T helper 17 cells and IL-17 production observed in inadequate responders to TNFi in patients with RA.^26^,^27^ Genovese et al. investigated the safety and efficacy of ABT-122, a dual variable domain immunoglobulin targeting human TNF and IL-17A, compared to TNFi (adalimumab) combined with MTX in both arms in patients with active RA (*n*=222) in a phase II RCT.^24^ Over the 12-week study period, dual inhibition of TNF and IL-17A with ABT-122 produced a safety profile consistent with that of adalimumab, with no serious infections or systemic hypersensitivity reactions reported with ABT-122. The efficacy of ABT-122 was not meaningfully different from that of the standard dose of adalimumab in patients with RA receiving concomitant MTX, precluding further development of ABT-122 for the treatment of RA. Glatt et al. further investigated the efficacy and safety of enhancing inadequate response to TNFi with IL-17i therapy.^25^ This proof of concept was tested in a phase IIa RCT that evaluated certolizumab pegol and bimekizumab, a novel monoclonal IgG1 antibody with dual inhibition of IL-17A and IL-17F approved for the treatment of psoriasis, in patients with moderate-to-severe RA with inadequate response to certolizumab pegol (*n*=159).^25^ At week 20, there was a greater reduction in DAS28 (CRP) (*primary outcome*), ACR50, and ACR70 (*secondary outcomes*) in the dual biologic treatment group compared with the certolizumab group. Safety-wise, there was a higher rate of treatment-emergent adverse effects in the dual biologic group compared to the certolizumab alone group, 78.8% (41/52) versus 59.3% (16/27), respectively. Severe adverse events were reported for one patient in each treatment group.

In summary, the studies discussed in this section are presented in Table 1. Although limited, the current evidence supports the potential usability of dual biologic therapy in resistant patients with RA with a concern for adverse safety profile for most tested combinations. A systemic review and meta-analysis on the safety of dual biologic therapy in patients with RA based on six studies concluded that there appears to be an increased risk of SAEs during the first 6–12 months of treatment, particularly in patients receiving the full dose of both biologics.^28^ Further research of this promising field is required to define the optimal biologic treatment combination and treatment candidates.

**Table 1:** Summary of Clinical Studies on Dual Biologic Therapy Indicated for Treatment of Rheumatoid Arthritis.

Author (Year), Country, ref.	Study Design	Dual Therapy (*n* Patients)	Control Arm (*n* Patients, if applicable)	Study Duration (mo)	Primary Study Outcome	Efficacy of Dual Biologic TX (Yes/No)	Safety Outcomes of Dual Biologic TX
Genovese (2004), USA^7^	RCT	Half-dosage ETN+ANA (*n*=81);Full-dosage ETN+ANA (*n*=81)	ETN (*n*=80)	6	Efficacy: ACR50 at 6 mo	No	Increased rate of SAEs
Weinblatt (2006), USA^8^	RCT	ABA+TNFi or ABA+ANA (*n*=103)	TNFi or ANA (*n*=64)	12	Safety	Post-hoc analysis: No	Increased rate of SAEs
Weinblatt (2007), USA^9^	RCT	ABA+ETN (*n*=85)	ETN (*n*=36)	12	Efficacy: ACR20 at 6 mo	No	Increased rate of SAEs
Blank (2009), Germany^10^	Retr.	RTX+ETN (*n*=6)	RTX (*n*=12)	8	Safety	Yes	Similar safety
Greenwald (2011), USA^11^	RCT	RTX (2×500 mg)+TNFi+MTX (*n*=33)	TNFi+MTX (*n*=18)	6	Safety	No	Similar safety
Rigby (2013), USA^12^	Open-label study	RTX (2×500 mg)+TNFi or ABA±DMARDs (*n*=176)	None	12	Safety	N.a.	Similar safety
Genovese (2018), Inter-national^24^	Phase II RCT	ABT-122 (*n*=166)	ADA (*n*=55)	3	Safety and efficacy: ACR20 at week 12	Similar efficacy	Similar safety
Glatt (2019), UK^25^	Phase IIa RCT	CTZ+BKZ (*n*=52)	CTZ (*n*=27)	5	Efficacy: DAS28 (CRP) at week 20 and safety	Yes	Increased rate of adverse events

ABA, abatacept; ABT-122, a dual variable domain immune-globulin targeting human TNF and IL-17A; ACR, American College of Rheumatology; ADA, adalimumab; ANA, anakinra; BKZ, bimekizumab; CRP, C-reactive protein; CTZ, certolizumab; DAS, disease activity score; DMARDs, disease-modifying antirheumatic drugs; ETN, etanercept; IL-17, interleukin 17; mo, months; MTX, methotrexate; N.a., not applicable; RCT, randomized controlled trial; Retr., retrospective; RTX, rituximab; SAEs, serious adverse events; TNFi, tumor necrosis factor inhibitor; TX, treatment; UK, United Kingdom; USA, United States of America.

### Dual Biologic Therapy Indicated for Treatment of Comorbidities

Osteoporosis and related fragility bone fractures represent a major source of morbidity in patients with RA.^15^ A chronic inflammatory state and disability contribute to development of osteoporosis in RA, further fostered by the use of glucocorticoids (glucocorticoid-induced osteoporosis, GIOP). The current treatment of osteoporosis relies on bisphosphonates as first-line drugs. Among the second-line treatments, denosumab offers an effective treatment for primary osteoporosis,^29^ GIOP,^30^ and as an adjunct therapeutic agent for RA. Denosumab is a biologic therapy composed of a fully human monoclonal antibody that inhibits bone resorption by inhibiting RANKL,^31^ shown to delay the progression of bone erosions and systemic bone loss in patients with RA treated with conventional synthetic DMARDs compared with placebo, with favorable safety profile.^32^–^34^ Several studies investigated the safety profile of dual therapy with bDMARDs and denosumab in patients with RA (Table 2).^17^–^19^,^35^ Curtis et al. evaluated infections among hospitalized RA patients treated with various bDMARDs in combination with denosumab (*n*=1,354) or zoledronic acid (*n*=4,460) as a comparator based on the US Medicare administrative claims database during 2006–2012.^18^ The rate of hospitalized infection (9–15/100 person-years), as well as type and sites of infection, was comparable between the two groups. The most common types of infections were genitourinary, sepsis, pneumonia, and skin or soft tissue infections. This study should be interpreted with caution due to a relatively short follow-up (slightly more than 6 months) in both exposure groups. Hasegawa et al. reported a single-center retrospective analysis of RA patients treated with various bDMARDs and denosumab (*n*=40) compared to age, gender, and disease characteristics-matched patients (*n*=40) followed for one year.^17^

**Table 2:** Summary of Observational Studies on Dual Biologic Therapy with Denosumab Indicated for Treatment of Osteoporosis as a Comorbidity of Rheumatoid Arthritis.

Author (Year), Country	Study Design	Dual Therapy (*n* Patients)	Control Arm (*n* Patients, if applicable)	Primary Study Outcome	Safety Outcomes of Dual Biologic TX
Curtis (2015), USA^18^	Retrospective Medicare database analysis	Various bDMARDs+DEN (*n*=1354)	Various bDMARDs+ zoledronic acid (*n*=4460)	Hospitalized infection rate	No increase of severe infections rate
Hasegawa (2016), Japan^17^	Retrospective case-control single-center study	Various bDMARDs+DEN (*n*=40)	Various bDMARDs (*n*=40)	Radiographic progression (modified Sharp erosion score)	No increase of infections and serious adverse events rate
Lau (2018), Canada^19^	Retrospective analysis of two rheumatology practices	Various bDMARDs+DEN (*n*=102)	Various bDMARDs (*n*=206)	Serious or opportunistic infection rate	Low rate of serious and opportunistic infections in both groups
Mirzaei (2021), Iran^35^	Retrospective case-control study	Various bDMARDs+DEN (*n*=40)	Various bDMARDs (*n*=44)	Rate of infections	Low rate of infections in both groups

bDMARD, biologic disease-modifying antirheumatic drug; DEN, denosumab; TX, treatment; USA, United States of America.

Concurrent use of denosumab was efficacious in inhibiting structural damage without increasing adverse events. Lau et al. reported a real-world experience of RA patients treated with various bDMARDs and denosumab (*n*=102) compared to patients treated with bDMARD alone (*n*=206) with a consistently favorable safety profile, i.e. low rate of serious or opportunistic infections, in both groups.^19^ Notably, in this study there was a low number of adverse events in both treatment groups, potentially attributed to a small sample size and short follow-up. Mirzaei et al. reported a small case control study of female patients with RA treated with various bDMARDs and denosumab (*n*=40) compared to those treated with bDMARD alone (*n*=44).^35^ In line with the previous studies, the infection rate was 4.5% in both groups, none of which required a hospitalization. In summary, current real-world based experience related to the use of denosumab concurrently with bDMARDs in patients with RA suggests a favorable safety profile of this combination.

Data on dual biologic therapy use for other than osteoporosis comorbidities derive mainly from case reports. A 69-year-old man with seropositive RA and severe eosinophilic asthma was successfully treated with a combination of golimumab and benralizumab, a humanized IL-5Ra monoclonal antibody.^36^ A 64-year-old female patient with RA and chronic spontaneous urticaria was treated with a combination of etanercept and omalizumab, a humanized monoclonal IgE antibody, with good results and no adverse effects.^37^ Another publication concisely reported on an RA patient successfully treated with a combination of abatacept and dupilumab, a human IL-4Ra monoclonal antibody, administered for severe atopic dermatitis without significant adverse events.^38^ These cases imply a potential therapeutic benefit of dual biologic therapies for refractory comorbidities, while the safety profile should be further evaluated in clinical trials.

## PSORIATIC ARTHRITIS

Multiple biologic therapies are currently available to treat PsA, yet refractory forms of the disease are not uncommon.^4^ Dual biologic therapy represents a promising therapeutic option for these cases based on the synergistic effect of the simultaneous targeting of different inflammatory pathways. Differently from RA, no clinical trials investigated the efficacy and safety of this treatment approach in PsA. Thibodeaux et al. summarized nine successful cases of dual biologic therapy use in PsA, including the following combinations: IL-12/23i (ustekinumab)+TNFi, IL-23i (guselkumab)+TNFi, and IL-17i (secukinumab)+TNFi.^14^ Several adverse events were noted in the above case reports. A 62-year-old patient with a background of metabolic syndrome experienced a cardiovascular event on treatment with etanercept and ustekinumab, without clear causality to the treatment.^39^ Under the same regimen, two infectious complications were reported: relapsing herpes zoster that was controlled after the reduction of etanercept dose, and a retrotonsillar abscess treated by incision and intravenous antibiotics.^13^ Successful dual therapy of PsA and concomitant severe atopic dermatitis with secukinumab and dupilumab, without adverse effects, was also reported.^40^ Haberman et al. reported on induction of remission in a biologic-naïve patient with severe psoriasis and PsA with dual combination therapy of TNFi (adalimumab) and ustekinumab, without side effects.^41^ This patient also underwent COVID-19 infection without complications. Notably, Metyas et al. reported a retrospective study of refractory PsA patients (*n*=22) treated with biologics and apremilast, a phosphodiesterase 4 inhibitor, added to the biologic regimen.^42^ Out of 22 patients, six patients developed side effects (nausea, diarrhea, weight loss, and abdominal pain), none of which caused discontinuation of therapy.

## CONCLUSION

Major advances in development of novel therapeutics for inflammatory arthropathies have led to improved clinical outcomes in a significant proportion of patients. Despite this progress, there remains an unmet clinical need in patients with refractory disease. Dual biologic therapy targeting different inflammatory pathways represents a promising therapeutic option for inflammatory diseases of complex and heterogeneous pathophysiology. There are ongoing pharmaceutical efforts to pursue this approach, including development of bispecific antibodies, such as ABT-122, TNFi, and IL-17i constructs, and conducting clinical trials on efficacy of dual biologic therapy not only in rheumatic diseases but also in the field of inflammatory bowel diseases.^43^ Although the first clinical trials in the field raised a concern regarding the safety of combined biologic therapy in patients with RA,^7^,^8^ later trials demonstrated a positive trend of additive clinical efficacy of dual biologic treatment with a favorable safety profile.^11^,^25^

Dual biologic therapy has been increasingly used not only for refractory patients but for treatment of comorbidities. Retrospective data on the combination of bDMARDs and denosumab in patients with RA consistently showed a favorable safety profile of this combination.^18^,^19^

To date, many questions remain unanswered regarding this novel field of therapy, including the choice of the most efficacious anti-cytokine biologics, along with the most appropriate timing, sequence, frequency, and duration of treatment. A potential strategy to decrease adverse events would be to use a lower dose of each therapy and consider asynchronous use of biologics with a loading dose of one drug followed by a sequential administration of another drug.

In summary, evidence for dual biologic treatment with anti-cytokine biologics remains limited, yet promising results are available in a number of clinical trials and real-world data. Along the rapidly evolving field of IMD treatment, dual therapy may constitute an efficacious and safe add-on treatment to biologic therapy, but properly conducted clinical investigations are needed. In the meantime, dual biologic therapy used by physicians’ discretion require close monitoring of patients with an emphasis on the safety profile.

## Abbreviations

ACR: American College of Rheumatology
bDMARDs: biologic disease-modifying antirheumatic drugs
IL: interleukin
IMD: immune-mediated diseases
MTX: methotrexate
PsA: psoriatic arthritis
RA: rheumatoid arthritis
RANK: receptor activator of nuclear factor kappa-B
RCT: randomized controlled trial
SAE: serious adverse event
TNFi: tumor necrosis factor inhibitor.
